# Isoform specific knockdown of the ETS transcription factor Pointed in *Drosophila* S2 cells

**DOI:** 10.17912/micropub.biology.000731

**Published:** 2023-05-23

**Authors:** Pavithra Vivekanand

**Affiliations:** 1 Biology Department, Susquehanna University, Selinsgrove, PA, USA

## Abstract

Alternate splicing of the *pointed* ( *pnt* ) gene locus produces two major isoforms, PntP1 and PntP2. Understanding their individual contributions to key developmental processes and identification of their genome-wide transcriptional targets has been hampered by a number of factors including their essential roles during embryonic development, and co-expression in several tissues. siRNAs were designed to target isoform-specific exons that code for the unique N-terminal region of either PntP1 or PntP2. The efficacy and specificity of the siRNAs were examined by co-transfection of isoform specific siRNAs with plasmids encoding epitope tagged PntP1 or PntP2 in *Drosophila* S2 cells. All P1-specific siRNAs were demonstrated to knockdown PntP1 protein level to greater than 95%, while having nominal impact on PntP2 level. Similarly, PntP2 siRNAs while ineffective at eliminating PntP1, were shown to reduce PntP2 protein level by 87-99%.

**Figure 1. Effect of isoform specific siRNAs on the expression levels of PntP1 and PntP2:**
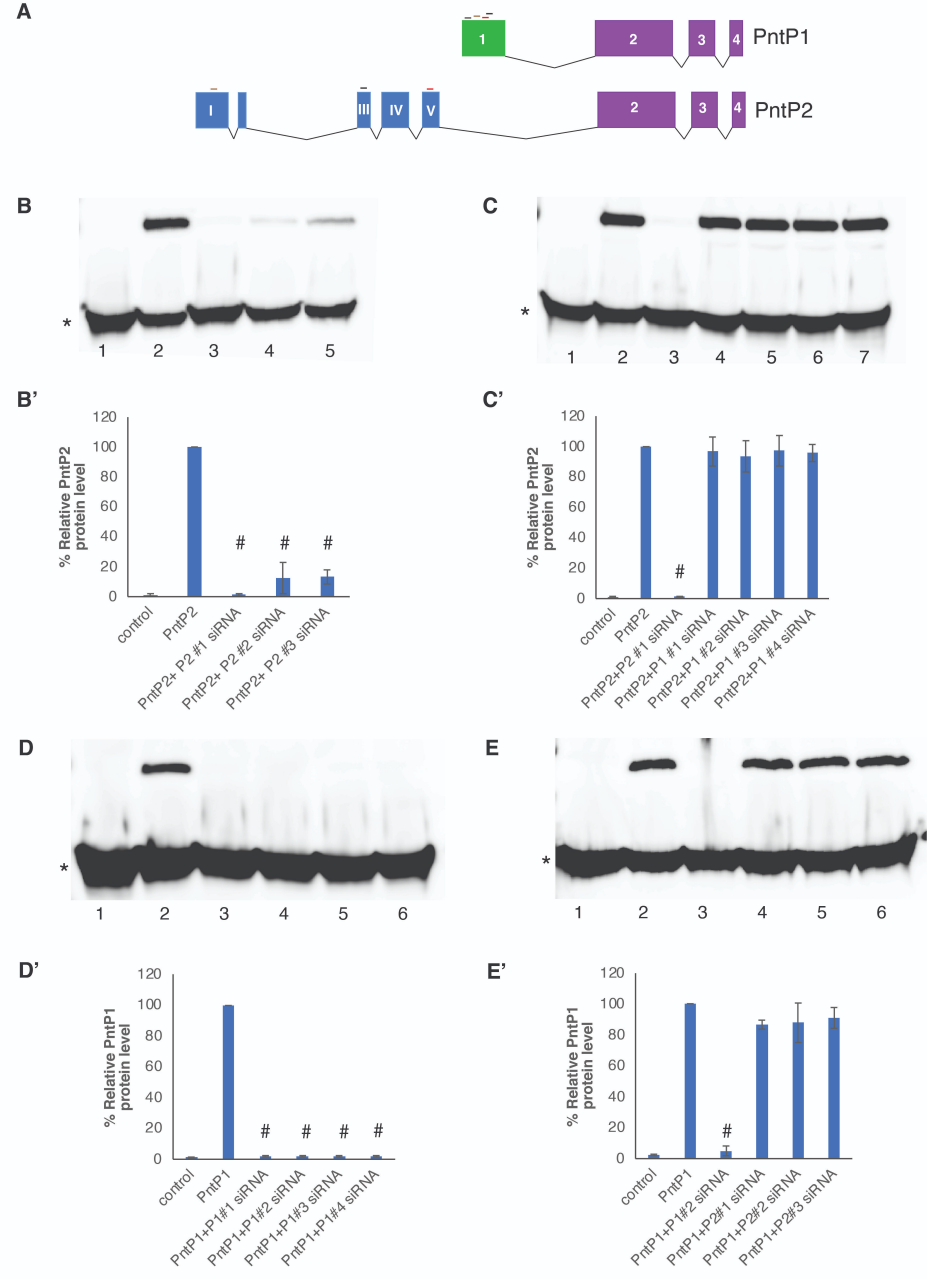
(A) Schematic representation of the exons that code for the PntP1 and PntP2 isoforms. Exons 2-4 are common to both isoforms, while exon 1 and exons I-V are unique for PntP1 and PntP2, respectively. The location of siRNA binding sites is indicated by horizontal lines above the exons. (B-E) *Drosophila* S2 cells were transfected with either Myc-PntP2 (B-C) or Flag-PntP1 expressing plasmid DNA (D-E) in the absence or presence of 20 nM of P1 or P2-specific siRNAs for 48 hours. (B, B’) PntP2 expression is absent in control cells that were not transfected with pPacPntP2 plasmid DNA (lane 1). PntP2 is expressed in cells transfected with plasmid DNA only (lane 2), while co-expression of P2 siRNAs results in a significant decrease in PntP2 expression level (lanes 3-5). (C, C’) Expression of PntP2 is unaffected by co-expression of P1 siRNAs (lanes 4-7), while co-expression of P2 siRNA (lane 3) decreases PntP2 expression level to that observed in untransfected control cells (lane 1). (D, D’) PntP1 is expressed in cells transfected with plasmid DNA only (lane 2) compared to untransfected control cells (lane 1), while co-expression of P1 siRNAs results in decrease in PntP1 expression level (lanes 3-6). (E, E’) P2-specific siRNAs have a minimal effect of the expression of PntP1 (lanes 4-6) when compared to co-expression of P1 siRNA (lane 3). Tubulin staining (indicated by *) was used as a loading control. Band intensity for PntP1, and PntP2 was quantified using Image J and normalized to the α-tubulin band in the corresponding lane. Data is expressed as a percentage with respect to cells transfected with plasmid DNA only, and reflects the average +/- SEM of 3 independent experiments. # P<0.0001 compared to cells transfected with either PntP1 or PntP2 DNA only.

## Description

Pointed (Pnt), an ETS family transcription factor, is a nuclear effector of the Receptor Tyrosine Kinase (RTK)/Ras/Mitogen-Activated Protein Kinase (MAPK) signaling pathway (Brunner et al. 1994; O'Neill et al. 1994; Gabay et al. 1996). Pnt function is critical for the differentiation of several cell types during embryonic and post-embryonic development (Scholz et al. 1993; O'Neill et al. 1994; Gabay et al. 1996; Jin et al. 2015; Vivekanand 2018). Transcription from alternate promoters and differential splicing produce two major isoforms of *pnt* , *pntP1* and *pntP2* (Klambt 1993; Scholz et al. 1993) *.* As a result, PntP1 and PntP2 have unique N-terminal regions. However, the isoforms possess identical C-terminal regions including the ETS DNA binding domain, and have been proposed to activate the same target genes. PntP1 does not require MAPK dependent phosphorylation and is a constitutive transcriptional activator, whereas, PntP2, like its vertebrate orthologs ETS1 and ETS2, must be phosphorylated by MAPK to be fully functional (Brunner et al. 1994; O'Neill et al. 1994; Albagli et al. 1996; Yang et al. 1996).

Since Pnt is a key transcription factor that mediates the effects of the RTK signaling pathway in several developmental tissues, understanding its function in post-embryonic development has necessitated the generation of mitotic clones. While PntP1 and PntP2 are predicted to regulate the expression levels of the same transcriptional targets, whether they do so *in vivo* has not been extensively investigated. This issue could be addressed using RNA interference (RNAi) to knockdown individual Pnt isoforms followed by RNA-seq analysis to identify genome-wide transcriptional targets. Such knockdown could be accomplished in the tissue of interest by crossing transgenic RNAi lines with tissue-specific GAL4 drivers, thereby circumventing the lethality exhibited by loss-of-function null alleles. RNAi lines targeting Pnt have been generated by the Transgenic RNAi Project (TRiP) and the Vienna Drosophila Resource Center (VDRC) (Dietzl et al. 2007; Perkins et al. 2015) . However, these RNAi lines target the shared C-terminal region and therefore affect the expression levels of all Pnt isoforms.

The goal of this study was to investigate whether isoform-specific siRNAs could be designed to mediate the knockdown of the intended Pnt isoform without impacting the expression levels of the untargeted isoform. To accomplish this, multiple siRNAs were designed to target the unique N-terminal region of either PntP1 or PntP2. Given the lack of antibodies that can be used to reliably detect the individual Pnt isoforms, *Drosophila* S2 cells were transfected with either Myc-tagged PntP2 (MycPntP2) or Flag-tagged PntP1 (FlagPntP1) expressing plasmids. MycPntP2 was expressed in S2 cells transfected with the PntP2 expressing plasmid but not in untransfected control cells ( Fig. 1B, lane 1 versus 2). ­­Co-transfection of P2-specific siRNAs with the PntP2-expressing plasmid, resulted in significant reductions in MycPntP2 expression levels for all three siRNAs ( Fig. 1B lane 2 versus lanes 3-5). Co-transfection of P2 siRNA #1 reduced MycPntP2 expression levels to that observed in untransfected control cells, while co-expression of either P2 siRNA #2 or P2 siRNA #3 resulted in reduction of the protein level to 13% of that observed in cells transfected with only the MycPntP2 plasmid ( Fig. 1B, B ’ lane 2 versus lanes 3-5). Contrasting the ability of the P2 specific siRNAs to reduce PntP2 levels, co-transfection of P1 specific siRNAs had no impact on MycPntP2 expression levels. Four different P1 specific siRNAs were tested, resulting in only a 3-6% reduction in MycPntP2 protein levels ( Fig. 1C, C ’ lane 2 versus lanes 4-7). To test the efficacy of P1-specific siRNAs in reducing PntP1 protein levels, S2 cells were transfected with Flag-PntP1 expressing plasmid. All four siRNAs targeting PntP1 reduced protein levels to that observed in untransfected control cells ( Fig. 1D, D ’ lane 2 versus lanes 3-6). Lastly, the specificity of P2-specific siRNAs was evaluated by examining their ability to knockdown the expression of PntP1 levels. Co-expression of the three P2 targeting siRNAs with Flag-PntP1 expressing plasmid resulted in a minimal reduction (9-13%) in PntP1 protein levels, compared to the 95% reduction brought about by co-transfection of a P1 specific siRNA (Fig 1E, E’ lane 3 versus lanes 4-6). Taken together, these results demonstrate that the PntP1 and PntP2 siRNAs were isoform specific and had minimal impact on the protein levels of the untargeted Pnt isoform.

Eukaryotic genomes have been shown to employ alternate splicing to increase the protein encoding capacity. Additionally, the co-expression of multiple protein isoforms can make it challenging to elucidate their individual contributions to the regulation of key developmental processes. While there have been several papers documenting the use of RNAi for isoform-specific knockdown in mammalian cells (Kisielow et al. 2002; Zhang et al. 2004; Goldberg and Sharp 2012) , there have been limited investigations on the feasibility of this approach in *Drosophila* . Celotto and Graveley (2002) used long dsRNAs that were designed to target mutually exclusive exon 4 variants to perform the isoform-specific knockdown of *Dscam* mRNA in *Drosophila***** cultured cells (Celotto and Graveley 2002) . Subsequent studies demonstrated that transgenes expressing dsRNA were able to eliminate isoforms of either the *Ecdysone Receptor* ( *EcR* ), or the *Dscam* gene, during larval stages (Roignant et al. 2003; Shi et al. 2007) . The *EcR* gene codes for three isoforms, EcR-A, EcR-B1 and EcR-B2, which have unique N-terminal domains. Accordingly, the researchers designed long dsRNA to target the unique N-terminal exon of either EcR-A or EcR-B1 to perform isoform-specific knockdown of EcR, without affecting the expression levels of the untargeted isoform (Roignant et al. 2003) . Similarly, isoform-specific knockdown of the exon that codes for alterative transmembrane (TM) regions of Dscam variants was performed, revealing the unique role of the Dscam TM variants in either dendritic elaboration or axonal arborization (Shi et al. 2007) . Therefore, isoform-specific knockdown of transcripts might be an effective strategy to uncover novel regulatory or developmental roles for co-expressed mRNAs.

In this study, I have demonstrated that siRNAs targeting unique sequences can be used to perform isoform-specific knockdown of PntP1 or PntP2 in *Drosophila* cultured cells. Recently, the role of a novel Pnt isoform, called *pntP3* was uncovered during photoreceptor specification (Wu et al. 2020) . *pntP3* is encoded by an N-terminal exon that is unique from that of either *pntP1* or *pntP2* . *pntP3* however shares the rest of the exons with *pntP2* including the exons that code for the SAM and the ETS domains. The isoform-specific N-terminal region of PntP1 is specified by a single exon, exon 1, while the N-terminal region of PntP2 is produced from 5 different exons, exons I-V, of which exons III-V are shared with PntP3 (Klambt 1993; Scholz et al. 1993; Wu et al. 2020). All 4 PntP1-specific siRNAs target exon 1 of PntP1, while the PntP2-specific siRNAs target exon I, III, and V. P2 siRNA #3 therefore uniquely targets the PntP2 isoform, while P2 siRNA #1 and #2 target both PntP2 and PntP3. Given PntP1 and PntP2 are co-expressed in certain tissues such as the intestinal stem cells, somatic follicle cells and the eye (Morimoto et al. 1996; Shwartz et al. 2013; Jin et al. 2015) , the ability to perform isoform-specific knockdown in these tissues would provide valuable insight into their individual functions. During *Drosophila* eye development, PntP3 was demonstrated to function redundantly with PntP2 for the specification of a subset of photoreceptors, and to activate PntP1 (Wu et al. 2020) . Given this added complexity, and the transcriptional feedback loops between the Pnt isoforms, it is imperative to perform isoform specific knockdown in different developmental tissues, and to exercise caution when interpreting the individual contributions of the different Pnt isoforms. Looking forward, the information provided by this investigation can be used to generate isoform-specific transgenic RNAi lines using either the VALIUM20 or WALIUM20 vectors for tissue, and isoform-specific knockdown of the different Pnt isoforms (Ni et al. 2008; Ni et al. 2011) .

## Methods

***Cell Line***

The S2-DRSC cell line was purchased from the Drosophila Genome Resource Center (DGRC Stock 181; https://dgrc.bio.indiana.edu//stock/181 ; RRID:CVCL_Z992) and cultured in Schneider's medium supplemented with 10% FBS and Penicillin/Streptomycin at 25°C.

***siRNA Sequences***

siRNAs were designed to the unique N-terminal sequences of PntP1 or PntP2 using IDT DNA technologies design tool. 10 mM siRNA stocks were made by adding Nuclease-free duplex buffer, vortexing briefly, and heating to 95°C for 2 minutes. The duplex siRNAs were allowed to cool at room temperature, aliquoted and stored at -20°C.

**Table:** 

Name	Sequence
PntP2 siRNA #1	5’- AAGGUCAACGAGGUACUGAAGGCAT -3’ 3’- CGUUCCAGUUGCUCCAUGACUUCCGUA- 5’
PntP2 siRNA #2	5’- CGAGAAACCAAAUGAGGAUAUUGTG -3’ 3’-ACGCUCUUUGGUUUACUCCUAUAACAC -5’
PntP2 siRNA #3	5’- GUCCGUCGAUACUUAGCCAAUUGAA -3’ 3’- GUCAGGCAGCUAUGAAUCGGUUAACUU -5’
PntP1 siRNA #1	5’- ACUACAACAUGGUCCUGCAAAGCTA -3’ 3’- GUUGAUGUUGUACCAGGACGUUUCGAU -5’
PntP1 siRNA #2	5’- GUCCUGCAAAGCUAUGAGAACUATC -3’ 3’- ACCAGGACGUUUCGAUACUCUUGAUAG- 5’
PntP1 siRNA #3	5’- AGUGAAUGCCAGCAUAAUCAGUGCC-3’ 3’-AAUCACUUACGGUCGUAUUAGUCACGG-5’
PntP1 siRNA #4	5’- GCAUCUCAAUCAGAGUCACUUCACC-3’ 3’-CUCGUAGAGUUAGUCUCAGUGAAGUGG-5’

***Transfection***

18-22 hours before transfection, S2 cells were plated at a density of 1 x 10 ^6^ cells/ml into 6-well plates. The cells were transfected with 2 μg of pPac-PntP2 (Act5c-MycPntP2) or pAFW-PntP1 (Act5c-FlagPntP1, gift from Dr. Zhu (Chen et al. 2021) ) plasmid DNA with or without 20nM of siRNAs using TransIT®-Insect Transfection Reagent (MIR-6100) according to the manufacturer’s instructions. The cells were incubated with the transfection mix for 48 hours to allow for protein expression.

***Protein Extractions and Western Blots***

Cells were lysed in 100 μL of 1X Laemmli sample buffer and the lysates were sonicated at 25 % amplitude for 15 seconds, followed by centrifugation at 4 °C for 10 minutes at 14000 rpm. Proteins were resolved using a 10 % SDS-PAGE and transferred onto a nitrocellulose membrane. Primary antibodies to detect Myc (71D10), and Flag (D6W5B) tagged Pnt were purchased from Cell Signaling Technology, while the anti-Tubulin antibody was purchased from DSHB (12G10). The nitrocellulose membrane was incubated overnight at 4°C with the relevant primary antibodies. All primary antibodies were used at 1:1000. The HRP conjugated anti-rabbit and anti-mouse antibodies were purchased from Jackson ImmunoResearch and used at 1:5000. Image J was used to quantify the expression levels of the PntP1 or PntP2 bands with respect to Tubulin and expressed as a percentage. Statistical significance was determined by one-way ANOVA.

## References

[R1] Albagli O, Klaes A, Ferreira E, Leprince D, Klämbt C (1996). Function of ets genes is conserved between vertebrates and Drosophila.. Mech Dev.

[R2] Brunner D, Dücker K, Oellers N, Hafen E, Scholz H, Klämbt C (1994). The ETS domain protein pointed-P2 is a target of MAP kinase in the sevenless signal transduction pathway.. Nature.

[R3] Celotto AM, Graveley BR (2002). Exon-specific RNAi: a tool for dissecting the functional relevance of alternative splicing.. RNA.

[R4] Chen R, Hou Y, Connell M, Zhu S (2021). Homeodomain protein Six4 prevents the generation of supernumerary Drosophila type II neuroblasts and premature differentiation of intermediate neural progenitors.. PLoS Genet.

[R5] Dietzl G, Chen D, Schnorrer F, Su KC, Barinova Y, Fellner M, Gasser B, Kinsey K, Oppel S, Scheiblauer S, Couto A, Marra V, Keleman K, Dickson BJ (2007). A genome-wide transgenic RNAi library for conditional gene inactivation in Drosophila.. Nature.

[R6] Gabay L, Scholz H, Golembo M, Klaes A, Shilo BZ, Klämbt C (1996). EGF receptor signaling induces pointed P1 transcription and inactivates Yan protein in the Drosophila embryonic ventral ectoderm.. Development.

[R7] Goldberg MS, Sharp PA (2012). Pyruvate kinase M2-specific siRNA induces apoptosis and tumor regression.. J Exp Med.

[R8] Jin Y, Ha N, Forés M, Xiang J, Gläßer C, Maldera J, Jiménez G, Edgar BA (2015). EGFR/Ras Signaling Controls Drosophila Intestinal Stem Cell Proliferation via Capicua-Regulated Genes.. PLoS Genet.

[R9] Kisielow M, Kleiner S, Nagasawa M, Faisal A, Nagamine Y (2002). Isoform-specific knockdown and expression of adaptor protein ShcA using small interfering RNA.. Biochem J.

[R10] Klämbt C (1993). The Drosophila gene pointed encodes two ETS-like proteins which are involved in the development of the midline glial cells.. Development.

[R11] Morimoto AM, Jordan KC, Tietze K, Britton JS, O'Neill EM, Ruohola-Baker H (1996). Pointed, an ETS domain transcription factor, negatively regulates the EGF receptor pathway in Drosophila oogenesis.. Development.

[R12] Ni JQ, Markstein M, Binari R, Pfeiffer B, Liu LP, Villalta C, Booker M, Perkins L, Perrimon N (2007). Vector and parameters for targeted transgenic RNA interference in Drosophila melanogaster.. Nat Methods.

[R13] Ni JQ, Zhou R, Czech B, Liu LP, Holderbaum L, Yang-Zhou D, Shim HS, Tao R, Handler D, Karpowicz P, Binari R, Booker M, Brennecke J, Perkins LA, Hannon GJ, Perrimon N (2011). A genome-scale shRNA resource for transgenic RNAi in Drosophila.. Nat Methods.

[R14] O'Neill EM, Rebay I, Tjian R, Rubin GM (1994). The activities of two Ets-related transcription factors required for Drosophila eye development are modulated by the Ras/MAPK pathway.. Cell.

[R15] Perkins LA, Holderbaum L, Tao R, Hu Y, Sopko R, McCall K, Yang-Zhou D, Flockhart I, Binari R, Shim HS, Miller A, Housden A, Foos M, Randkelv S, Kelley C, Namgyal P, Villalta C, Liu LP, Jiang X, Huan-Huan Q, Wang X, Fujiyama A, Toyoda A, Ayers K, Blum A, Czech B, Neumuller R, Yan D, Cavallaro A, Hibbard K, Hall D, Cooley L, Hannon GJ, Lehmann R, Parks A, Mohr SE, Ueda R, Kondo S, Ni JQ, Perrimon N (2015). The Transgenic RNAi Project at Harvard Medical School: Resources and Validation.. Genetics.

[R16] Roignant JY, Carré C, Mugat B, Szymczak D, Lepesant JA, Antoniewski C (2003). Absence of transitive and systemic pathways allows cell-specific and isoform-specific RNAi in Drosophila.. RNA.

[R17] Scholz H, Deatrick J, Klaes A, Klämbt C (1993). Genetic dissection of pointed, a Drosophila gene encoding two ETS-related proteins.. Genetics.

[R18] Shi L, Yu HH, Yang JS, Lee T (2007). Specific Drosophila Dscam juxtamembrane variants control dendritic elaboration and axonal arborization.. J Neurosci.

[R19] Shwartz A, Yogev S, Schejter ED, Shilo BZ (2013). Sequential activation of ETS proteins provides a sustained transcriptional response to EGFR signaling.. Development.

[R20] Vivekanand P (2018). Lessons from Drosophila Pointed, an ETS family transcription factor and key nuclear effector of the RTK signaling pathway.. Genesis.

[R21] Wu C, Boisclair Lachance JF, Ludwig MZ, Rebay I (2020). A context-dependent bifurcation in the Pointed transcriptional effector network contributes specificity and robustness to retinal cell fate acquisition.. PLoS Genet.

[R22] Yang BS, Hauser CA, Henkel G, Colman MS, Van Beveren C, Stacey KJ, Hume DA, Maki RA, Ostrowski MC (1996). Ras-mediated phosphorylation of a conserved threonine residue enhances the transactivation activities of c-Ets1 and c-Ets2.. Mol Cell Biol.

[R23] Zhang L, Yang N, Liang S, Barchetti A, Vezzani C, Huang J, O'Brien-Jenkins A, Rubin SC, Coukos G (2004). RNA interference: a potential strategy for isoform-specific phosphatidylinositol 3-kinase targeted therapy in ovarian cancer.. Cancer Biol Ther.

